# Combining Pixel Swapping and Simulated Annealing for Land Cover Mapping

**DOI:** 10.3390/s20051503

**Published:** 2020-03-09

**Authors:** Lijuan Su, Yue Xu, Yan Yuan, Jingyi Yang

**Affiliations:** Key Laboratory of Precision Opto-mechatronics Technology Sponsored by Ministry of Education, School of Instrumentation Science and Opto-electronics Engineering, Beihang University, Beijing 100191, China; xuyue1995@buaa.edu.cn (Y.X.); YangJan@buaa.edu.cn (J.Y.)

**Keywords:** remote sensing image, subpixel mapping, simulated annealing algorithm, pixel-swapping algorithm

## Abstract

Mixed pixels commonly exist in low-resolution remote sensing images, and they are the key factors hindering the classification of land covers and high-precision mapping. To obtain the spatial information at the subpixel level, subpixel mapping (SPM) technologies, including the pixel-swapping algorithm (PSA), that use the unmixed proportions of various land covers and allocate subpixel land covers have been proposed. However, the PSA often falls into a local optimum solution. In this paper, we propose a SPM method, the PSA_MSA algorithm, that combines the PSA and the modified simulated annealing algorithm to find the global optimum solution. The modified simulated annealing algorithm swaps subpixels within a certain range to escape the local optimum solution. The method also optimizes all the mixed pixels in a randomized sequence to further improve the mapping accuracy. The experimental results demonstrate that the proposed PSA_MSA algorithm outperforms the existing PSA-based algorithms for SPM. The images with different spatial dependences are tested and the results show that the proposed algorithm is more suitable for images with high spatial autocorrelation. In addition, the effect of proportion error is analyzed by adding it in the experiments. The result shows that a higher proportion error rate leads to larger degradation of the subpixel mapping accuracy. Finally, the performance of PSA_MSA algorithm with different ranges of selection on subpixel-swapping is analyzed.

## 1. Introduction

Remote sensing technologies have been increasingly used in many areas, such as environmental monitoring, agricultural production, and resource exploration. The spatial resolution and spectral resolution of spectral imaging systems are mutually restricted. To obtain a higher spectral resolution, the spatial resolution of a spectral imaging system has to be sacrificed [1]. As a result, the spatial resolution of spectral imaging systems is lower than that of panchromatic imagers. In images with low spatial resolutions, one image pixel might include several different types of land cover [2,3,4,5,6]. These land covers have different spectral characteristics. A pixel containing only one type of land cover is called a pure pixel. Otherwise, it is called a mixed pixel. To obtain the end-member information, spectral unmixing algorithms that can determine the proportion of different land covers in the mixed pixels have been proposed, including the linear mixture model [7,8], the fuzzy c-means classification model [9,10], and the neural network model [11,12]. Although proportional images can show the proportions of different land cover types in mixed pixels and provide a better understanding of the features contained in the mixed pixels, they still cannot provide the spatial distributions of the determined classes.

However, in many applications, high spatial resolution images of land covers classifications are highly desired. To achieve high-resolution results, Atkinson [13] proposed the subpixel mapping (SPM) algorithm, which predicts subpixel spatial distributions of land covers by using the proportion information. The SPM uses the proportion information of land cover types to determine the spatial distributions of those land covers in low-resolution spectral images. Consequently, since SPM can obtain the locations of various land covers at the subpixel level, it can improve the spatial resolution of spectral images. Various SPM algorithms based on spatial dependence theory [14,15,16,17,18] and the neural network model [19,20,21,22,23,24] have been proposed in the past twenty years. Among them, the pixel-swapping algorithms (PSA) based on spatial dependence theory is the main algorithm in SPM due to its accurate and understandable properties. The PSA was first proposed by Atkinson [13,25] to maximize the spatial correlation of the subpixels by exchanging the classes of the subpixels. At present, research on the PSA is constantly proposed. Makido and Shortridge [26] discussed the influence of PSA settings on SPM. Luciani and Chen [27] tested the impact of the image and class structure of the input imagery on the accuracy of the outputs from the PSA. Their work validated the known positive correlation between spatial autocorrelation and PSA accuracy and demonstrated that the class proportion differences and mixed pixel quantities were positively and negatively related to PSA accuracy, respectively. Studies have also been performed to improve the mapping accuracy of the PSA. Mertens et al. [28] introduced a subpixel/pixel attraction model (SAM) that determines the spatial relationship between subpixels and pixels. Shen [29] used the PSA to optimize the output of the SAM to improve the mapping accuracy. Su et al. [30] proposed a method that combines the PSA and contouring approaches to enhance SPM. This method also considered the influence of the number of neighboring subpixels and the downscale factor on the accuracy of SPM. Li et al. [31] used plurality voting to select the class label for each subpixel from the multiple SPM outputs available. Peng et al. [32] applied the simulated annealing algorithm into the pixel-swapping algorithm to optimize the objective function (PSA_SA). Wu et al. [33] proposed a double-calculated spatial attraction model (DSAM) which applied symmetrical patterns to initialize the subpixel assignment and to optimize the reconstruction through subpixel exchanges. He also analyzed the influence of the reconstruction scale, image spatial resolution, and pixel spatial relationships on the SPM accuracy. These algorithms combined different algorithms to improve the mapping accuracy based on PSA but the problem of PSA easily settling for the local optimal solution [34] has not been fully addressed.

In order to improve this problem, a pixel-swapping algorithm based on the modified simulated annealing algorithm (PSA_MSA) is proposed in Section 2 of this paper. After adding the simulated annealing (SA) algorithm, the PSA_MSA algorithm only exchanges subpixels in a specific range. Simulation experiments have been performed to compare the mapping accuracy of the proposed PSA_MSA algorithm with the accuracy of other algorithms: PSA, PSA simulated annealing (PSA_SA), and DSAM. Among them, the PSA is the classical subpixel swapping algorithm, the PSA_SA is a PSA with SA algorithm and the DSAM is the latest algorithm based on subpixel swapping. The effect of the proportion of error on the accuracy of subpixel mapping is also discussed.

## 2. Materials and Methods

### 2.1. Principles of the PSA

**Figure 1:**
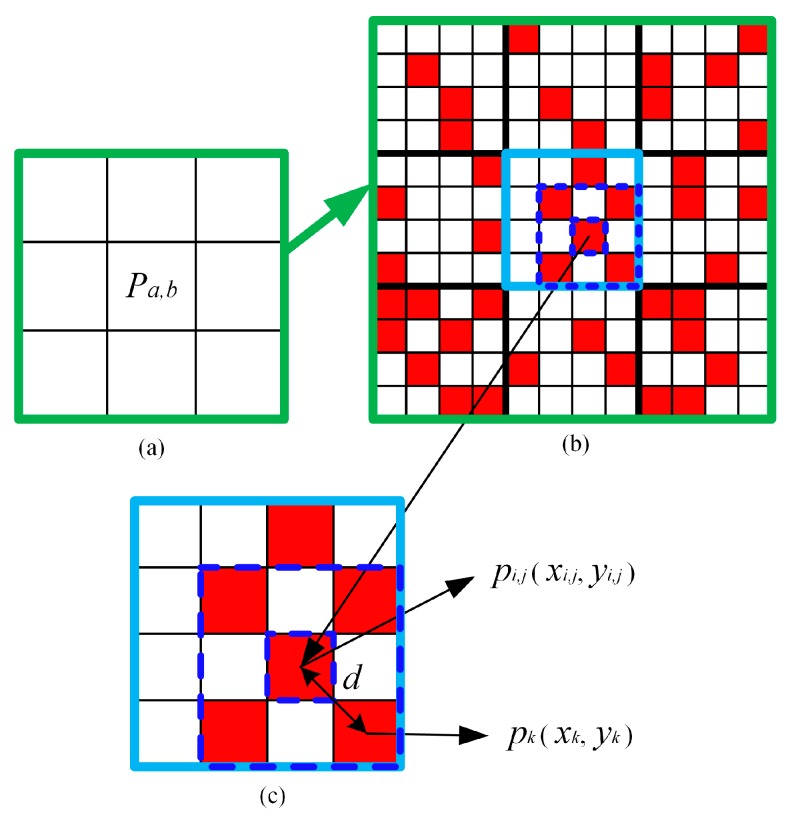
Spatial correlations of the subpixels in the pixel-swapping algorithm (PSA) (*S* = 4). (**a**) Mixed pixels, (**b**) high-resolution image, (**c**) subpixels in *P*_*a*,*b*_.

We begin with a brief introduction of the principle behind and the functions of the PSA. The PSA constantly exchanges classes of subpixels to ensure that the subpixels from the same object have the largest spatial correlation. The PSA consists of three steps. Some symbols are shown in Figure 1.

Step 1. Calculate the gravitational value which reflects the spatial correlation of a mixed pixel. For a subpixel *p*_*i*,*j*_ located at (*i*, *j*), its gravitational value can be calculated as follows:

(1) $$A_{p_{i,j}}=\sum_{k=1}^{N}\lambda_{k}z(p_{i,j},p_{k})$$

where $A_{p_{i,j}}$ is the gravitational value of *p*_*i*,*j*_, *N* is the number of neighboring subpixels, *λ_k_* is the weighting function of distance, and z(*p*_*i*,*j*_, *p_k_*) is the And operation function.

The total gravitational value of a mixed pixel *P*_*a*,*b*_ can be obtained by summing the gravitational values of all its subpixels *p*_*i*,*j*_.

(2) $$A_{P_{a,b}}=\sum_{i=1}^{s}\sum_{j=1}^{s}A_{p_{i,j}}$$

Step 2. Exchange the classes of the subpixels within the mixed pixel. For all subpixels of category 1, the subpixels with the lowest gravitational value are set to 0; for all subpixels of category 0, the subpixels with the lowest gravitational value are set to 1.

Step 3. Based on Equation (5), calculate the new total gravitational value of the mixed pixel. If the new gravitational value increases, then a subpixel-swapping result is retained. Otherwise, the locations of different land cover subpixels will return to the previous state.

Repeat step 1 to step 3 until the total gravitational value (Equation (5)) of the mixed pixel does not increase. The corresponding SPM results are considered to be the best localization results.

The current swapping method of the PSA is relatively simple, yet it is likely to fall into a local optimum solution of the objective function. To solve this problem, we introduce a SA algorithm to obtain the global optimum solution during subpixel swapping.

### 2.2. PSA with Simulated Annealing (SA)

The SA algorithm, which is used for solving optimization problems, was first proposed by Kirkpatrick et al. [35]. It is based on a strong analogy between the issue of solving optimization problems and the physical annealing of solids, which leads a system to its lowest energy state. Thus, the SA algorithm has the ability to escape from the local optimum solution [36,37].

A simulated annealing process includes the following steps: (1) select an objective function and set the starting temperature (simulated melting point), (2) simultaneously reduce the temperature and decrease the energy of the objective function, and (3) repeat step (2) until the target temperature reaches the ideal temperature and the objective function converges to the minimum value.

Peng 32 directly applied the conventional SA algorithm to the swapping process of the PSA, and we refer to this algorithm as the PSA_SA. Peng used the spatial correlation to calculate the objective function used in the SA algorithm. The spatial correlation coefficients are calculated as follows:

(3) $$A_{pi,j}=\sum_{k=1}^{N}\frac{\delta_{p_{i,j},p_{k}}}{N}$$

where *N* is the number of neighboring subpixels of each subpixel, while $\delta_{p_{i,j},\;p_{k}}$ is the And operation function.

The final target optimization function of one mixed pixel is the summation of the spatial correlation coefficient of each subpixel within this mixed pixel and is defined as:

(4) $$A_{P_{a,b}}=\sum_{i=1}^{S}\sum_{j=1}^{S}A_{pi,j}.$$

The subpixel swapping process of PSA_SA is as follows: Randomly exchange the classes of subpixels in a mixed pixel, and calculate the new target optimization function $A_{P_{a,b}}$′ and the corresponding Δ$A_{P_{a,b}}$=$A_{P_{a,b}}$′ − $A_{P_{a,b}}$. If Δ$A_{P_{a,b}}$ ≥ 0, the exchanged positions of subpixel land covers would be retained, and $A_{P_{a,b}}$ *=* $A_{P_{a,b}}$′. If Δ$A_{P_{a,b}}$ < 0, calculate the acceptance probability *ε* = exp(−$A_{P_{a,b}}$/*T*) at the current SA temperature *T*, and compare *ε* with a random number *r,* which is in the range of (0,1). If *ε* > *r*, then the exchanged result would also be retained. Otherwise, the location of subpixels will return to the previous state.

Unlike the PSA, the PSA_SA contains an added SA algorithm, which has shown a great tolerance to local optima convergence and is often called a global optimizer that accepts deteriorated solutions with a certain probability in subpixel swapping [38]. Thus, the PSA_SA should be able to help mixed pixels to get rid of the problem of the local minimum in the process of increasing their gravitational value through subpixel swapping. However, the constant random exchange in the SA algorithm makes it difficult to use the subpixels of various land cover types to establish a reasonable spatial distribution. Therefore, the accuracy of the PSA_SA is not ideal, which will be demonstrated in the experimental results in Section 3.

### 2.3. PSA Based on the Modified Simulated Annealing Algorithm (PSA_MSA)

According to Atkinson, the purpose of the PSA is to maximize the spatial dependence, which is “the likelihood that observations close together are more alike than those that are further apart” 25, for subpixels of different land covers in a mixed pixel. To achieve this goal, his proposed PSA swaps the subpixels corresponding to the two types of land covers that have the smallest gravitational values. Peng’s method merely swaps two randomly selected subpixels from all the subpixels of different land covers. Although this method considers the spatial dependence among the subpixels, the swapping range in this method is too wide and therefore cannot obtain sufficiently accurate results within a limited number of interactions.

**Figure 2:**
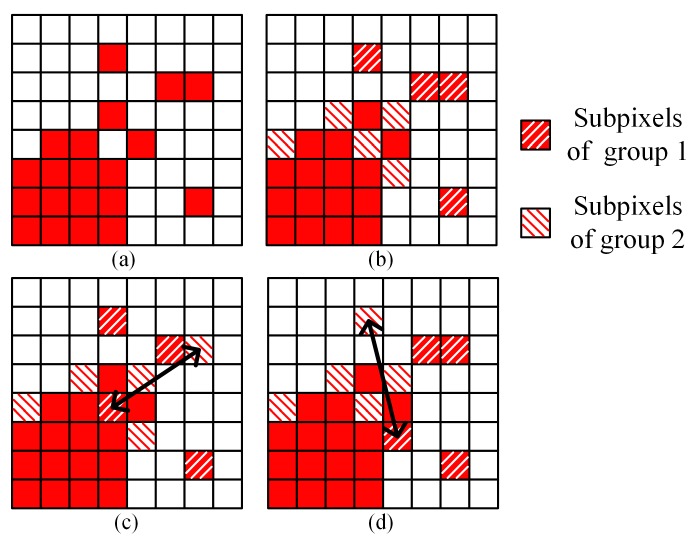
Interpretation of the PSA_MSA algorithm. (**a**) Original image, (**b**) randomly selected subpixels for swapping, (**c**) Swapping result 1, (**d**) Swapping result 2.

In this research, based on the spatial dependence principle, we propose a PSA based on the modified simulated annealing algorithm. Figure 2a shows a mixed pixel with two kinds of land cover. Several subpixels with small gravitational values in the corresponding land cover form a group, and two groups of these subpixels are shown as slashed blocks in Figure 2b. In the proposed method, the SA algorithm randomly selects only one pixel from each group and swaps their classes. Figure 2c,d show two possible swapping results. This method constrains the subpixels, which are randomly swapped by SA, to subpixels with small gravitational values within their own classes.

**Figure 3:**
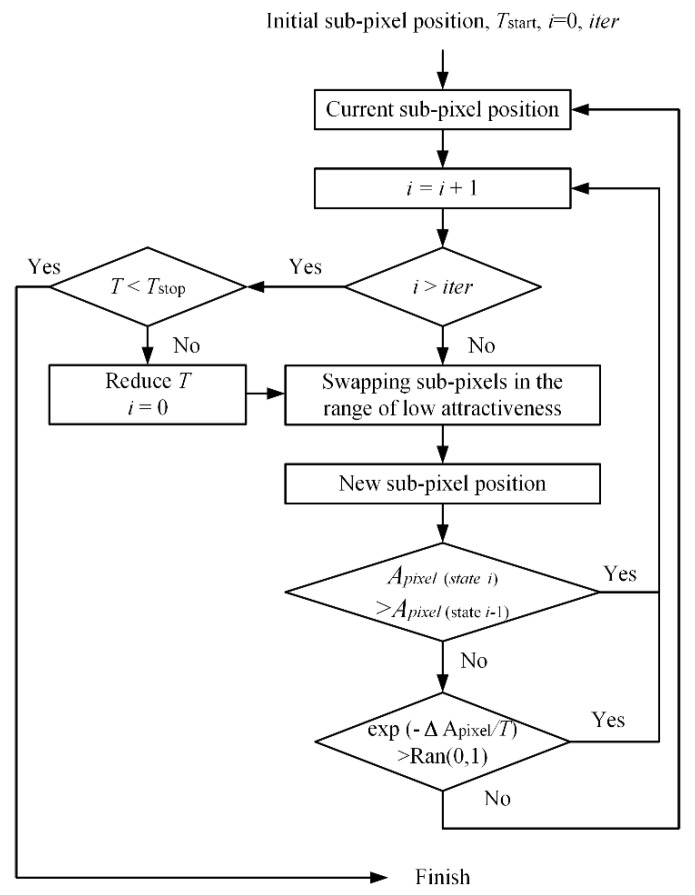
Flow chart of the modified simulated annealing algorithm.

The procedure of the proposed PSA_MSA algorithm following various steps: Figure 3 illustrates the process of optimizing mixed pixels from step 1 to step 3 in the form of a flow chart.

Step 1. Initialization

According to the proportions of land cover in the mixed pixels, the subpixels with corresponding classes are randomly allocated in each mixed pixel. Calculate the total attractiveness of each mixed pixel by using either Equation (2) or Equation (4). Equation (2) considers the distance between the central subpixel and neighboring subpixels as the weight function, while Equation (4) treats neighboring subpixels with the same weighting value. We denote the algorithms based on Equations (2) and (4) as PSA_MSA1 and PSA_MSA2, respectively.

In the SA algorithm, set the values of the parameters such as the attenuation factor (*a*), the number of iterations (*iter*), the initial temperature *T*_start_, and the final temperature *T*_stop_. The cooling function is defined by *T_q_*_+1_ *= T_q_* × *a*, where *q* is the number of cooling times which reflects the number of iterations and *T*_0_ = *T*_start_.

Step 2. Use the SA algorithm to randomly swap the selected subpixels

According to the principle of spatial relevance, we can only swap subpixels with low attractiveness. Therefore, a constraint is applied to the SA algorithm by selecting subpixels that need to be swapped with attractiveness values within a certain range.

The low attractiveness range of is defined as follows:

First, the subpixel gravitational values of two opposing classes are calculated separately. Here, we use M and N to represent two opposing classes. There are *l* subpixels in class M and *S* × *S* − *l* subpixels in class N.

Then, the gravitational values of M-class subpixels are sorted from smallest to largest: $A_{M_{1}}$ < $A_{M_{2}}$ < … < $A_{M_{u}}$ < … < $A_{M_{m}}$ (*m ≤ l*; some subpixels may have the same gravitational value). Moreover, the gravitational values of N-class subpixels are sorted from smallest to largest: $A_{N_{1}}$ < $A_{N_{2}}$ < … < $A_{N_{v}}$ < … < $A_{N_{n}}$ (*n* ≤ *S* × *S* − *l*). In this research, the low attractiveness range for class M is [0, $A_{M_{u}}$], and the low attractiveness range for class N is [0, $A_{N_{v}}$]. Then, the swapping process can be expressed by the following formula:

(5) $$\left\{ \begin{array}{l} \mathrm{randomly}\;\mathrm{select}\;p_{i,j}=N\;\{A_{p_{i,j}}\leq A_{M_{u}}|p_{i,j}=M\}\\ \mathrm{randomly}\;\mathrm{select}\;p_{i,j}=M\;\{A_{p_{i,j}}\leq A_{N_{v}}|p_{i,j}=N\} \end{array}\right.$$

where $A_{p_{i,j}}$ is the attractiveness of subpixel *p*_*i*,*j*_ calculated by either Equation (1) or (3).

Calculate the new total objective function values $A_{P_{a,b}}$′ and Δ*E* = $A_{P_{a,b}}$′ − $A_{P_{a,b}}$. If Δ*E >* 0, the new positions of the subpixels will be stored. If Δ*E* ≤ 0, compute the acceptance probability *ε* = exp($A_{P_{a,b}}$′ − $A_{P_{a,b}}$)/*T_q_* at the current MSA *T_q_*. If *ε* > *r*, where *r* = rand (0,1), the new position of the subpixels will still be accepted. Otherwise, the locations of the subpixels will return to the previous state.

Step 3. Cooling system

Once step 2 is accomplished, *i* = *i* + 1. If *i* = *iter*, the system will be cooled by the cooling function: *T_q_*_+1_ *_=_ T_q_* × *a*.

Repeat steps 2 and 3. The optimization of one mixed pixel will be stopped when *T_q_*_+1_ *< T*_stop_. All mixed pixels will be optimized via steps 1 to 3.

Step 4. Randomly optimize the mixed pixels a second time

**Figure 4:**
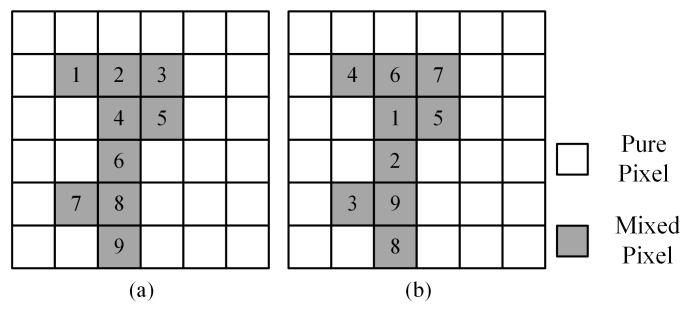
Different mixed pixel selection methods: (**a**) Sequential and (**b**) Stochastic.

The traditional PSA sequentially optimizes the mixed pixels, as shown in Figure 4a. However, the experimental results show that sequential optimization may limit the accuracy of SPM, which may be due to repeated one-directional optimization. To change the optimization direction, we randomly adjusted the order of the mixed pixels, as shown in Figure 4b and optimized the mixed pixels again following the same procedures described in steps 2 and 3. The experimental results in Section 3.4 show that randomly optimizing the mixed pixels again is better than sequentially optimizing them.

Step 5. Winner-takes-all strategy

Since the SA algorithm uses binary optimization, only one type of land cover is optimized at a time. In each iteration, only the subpixels of one land cover type are set to 1 (*p*_*i*,*j*_ = 1), and the *p*_*i*,*j*_ for all the other subpixels are set to 0. To optimize all the land covers, steps 1 to 4 are repeated. After all land covers are optimized with the initial input proportional image, the corresponding class-specific attractiveness of the subpixels is calculated. Thus, each subpixel stored the attractiveness values corresponding to different types of land covers. Comparing the attractiveness values of all kinds of land covers within a subpixel, the subpixel will be classified according the land cover that has the largest attractiveness value 15.

## 3. Experiments and Analysis

To evaluate the proposed PSA_MSA algorithm, four different types of experiments were performed. Considering that SPM is only an image processing technology for proportion images, all tested images were classified and processed from high-resolution images. A mean filter is used to degrade these high-resolution images into low-resolution images with mixed pixels. The size of the mean filter is set to *S* × *S*, where *S* is the reconstruction scale. Thus, other errors can be avoided when generating proportion images, so that the reconstruction results are reliable and the positioning accuracy of different SPM methods are true and authentic. In the first three experiments, the PSA, the PSA_SA, and the DSAM were selected for comparison. In the fourth experiment, the influence of errors on the process of proportion acquisition in the SPM_MSA algorithm is tested. In the last experiment, the impact of the random optimization procedure of the PSA_MSA algorithm is discussed.

The overall accuracy is evaluated in terms of the percentage of correctly classified subpixels (PCC) and the classic Kappa coefficient [39]. The larger the PCC and Kappa values are, the more subpixels that are correctly classified and the better the reconstruction result. According to Merterns, the improved PCC′ and Kappa′ “are identical to PCC and Kappa except that they are calculated only for mixed pixels” [40]. The subpixels in pure pixels all belong to the same class and will undoubtedly increase both values of PCC and Kappa. Thus, the improved PCC′ and Kappa′ can better reflect the performance of different SPM methods than the original PCC and Kappa, respectively. Therefore, we also use the improved PCC′ and Kappa′ to evaluate the performance.

### 3.1. Artificial Shapes

**Figure 5:**
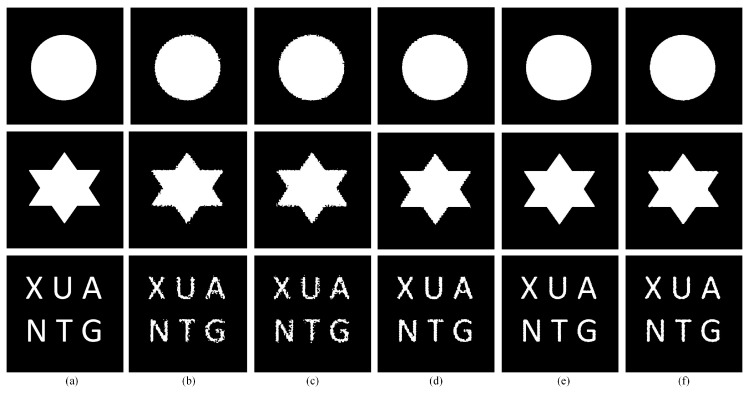
Comparison of the results from different SPM models for artificial shapes at a reconstruction scale of 10: (**a**) the reference image, (**b**) the PSA results, (**c**) the PSA_SA algorithm results, (**d**) the DSAM algorithm results, (**e**) the PSA_MSA1 algorithm results, and (**f**) the PSA_MSA2 algorithm results.

In the first experiment, three different artificial shapes, as shown in Figure 5a, were used to test the algorithms. The reconstruction scale *S* was set to 10. In the SPM method based on SA, the parameters are set as follows: *T*_start_ = 10 × *S* = 10 × 10, *iter* = 5, *a* = 0.8, and *T*_stop_ = 0.01. In the PSA_MSA model, *u* and *v*, which determine the range of subminimum attractiveness, were both set to 2. The results of the different algorithms are shown in Figure 5b–f. The corresponding evaluation criteria of the different methods are summarized in Table 1. It is obvious that the reconstruction results from directly using a conventional SA swapping algorithm are not as good as expected. This finding shows that the completely random character of the conventional SA algorithm does not fit the SPM problem very well. Comparing the results of the PSA and those obtained by the modified SA algorithms, the modified SA algorithms obtain much better results, regardless of the type of shape in the image: circle, star, or letters. The PSA_MSA1 and PSA_MSA2 algorithms are based on our MSA algorithm with objective functions given by Equation (2) and Equation (4), respectively. The performances of these two algorithms are relatively close. The resulting images with letters show that the objective function based on Equation (4) is more suitable for more sophisticated mixed pixels with finer geometrical details and shapes than is the objective function based on Equation (2).

### 3.2. Artificial Land Images

**Figure 6:**
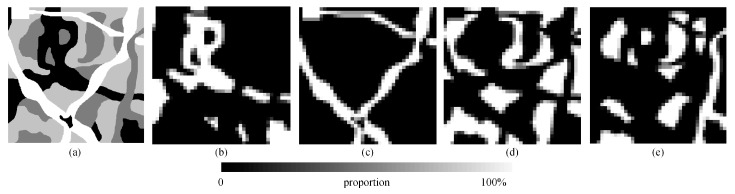
Proportional images of four classes with a scale of 12. (**a**) Reference image, (**b**) class 1, (**c**) class 2, (**d**) class 3, (**e**) class 4.

**Figure 7:**
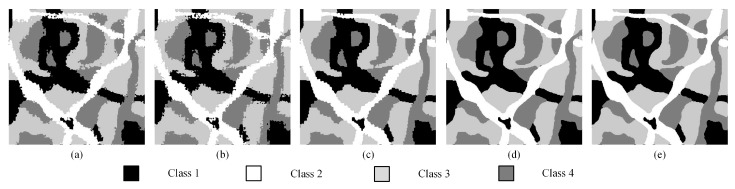
Comparison results of different SPM models for an artificial land image with a scale of 12. (**a**) PSA result, (**b**) PSA_SA result, (**c**) DSAM result, (**d**) PSA_MSA1 result, (**e**) PSA_MSA2 result.

To demonstrate the spatial positioning ability of our algorithm on SPM, we applied the above algorithms to an artificial image containing four land cover types. The 450 × 450 artificial land image is shown in Figure 6a. Different shapes and boundaries are included in the artificial land image. The reconstruction scales were set to 8, 10, and 12. The parameters of the SA-based algorithms are the same as in Section 3.1. Figure 7a–e show the mapping results of the PSA, the PSA_SA algorithm, the DSAM algorithm, the PSA_MSA1 algorithm, and the PSA_MSA2 models at *S* = 12. It is obvious that the PSA_MSA1 and the PSA_MSA2 models reflect the spatial resolution of different classes better than the other three models did.

The PCC, Kappa, improved PCC′, and Kappa′ values for different algorithms at the three reconstruction scales are summarized in Table 2. The larger the reconstruction scale is, the lower the accuracies of these methods because the mixed geometries are more complicated as the reconstruction scale increases; consequently, the uncertainty of the positioning process is inevitable.

At the same reconstruction scale, the PCC, Kappa, and improved PCC′, Kappa′ values from the PSA, the PSA_SA and DSAM algorithm were lower than the values from the PSA_MSA1 and the PSA_MSA2 algorithms, which illustrates that the PSA_MSA algorithm performed better than the other three algorithms. Taking *S* = 12 as an example, the improved Kappa′ value from the PSA_MSA1 and the PSA_MSA2 algorithms reached 94.77 and 94.99, respectively. The improved Kappa′ value from the PSA_MSA1 was 12.79%, which is 20.80% higher than that of the PSA and the PSA_SA algorithm. Additionally, it is 4.86% higher than that of DSAM as well. Meanwhile, the improved Kappa′ value from the PSA_MSA2 algorithm was 13.06%, which is 21.08% higher than that of the PSA and the PSA_SA algorithm. In addition, it is 5.10% higher than that of DSAM as well. Comparing the results of the PSA_MSA1 and the PSA_MSA2 algorithms, the PSA_MSA1 algorithm performs slightly better than the PSA_MSA2 algorithm at low reconstruction scales. As the reconstruction scale increases, the PSA_MSA2 algorithm performs slightly better than the PSA_MSA1 algorithm. This finding further confirms that Equation (4) is more suitable for low-resolution images with more sophisticated geometries than Equation (2).

**Figure 8:**
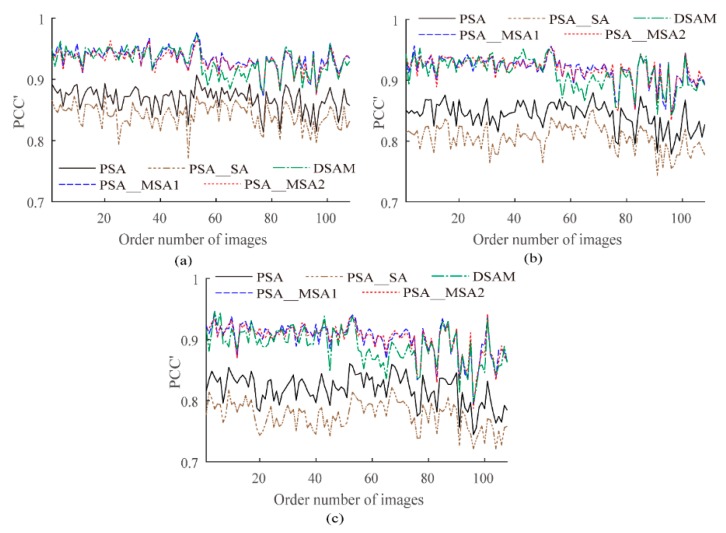
The improved PCC′ values (%) of different algorithms for the synthetic data set. (**a**) *S* = 8, (**b**) *S* = 10, (**c**) *S* = 12.

**Figure 9:**
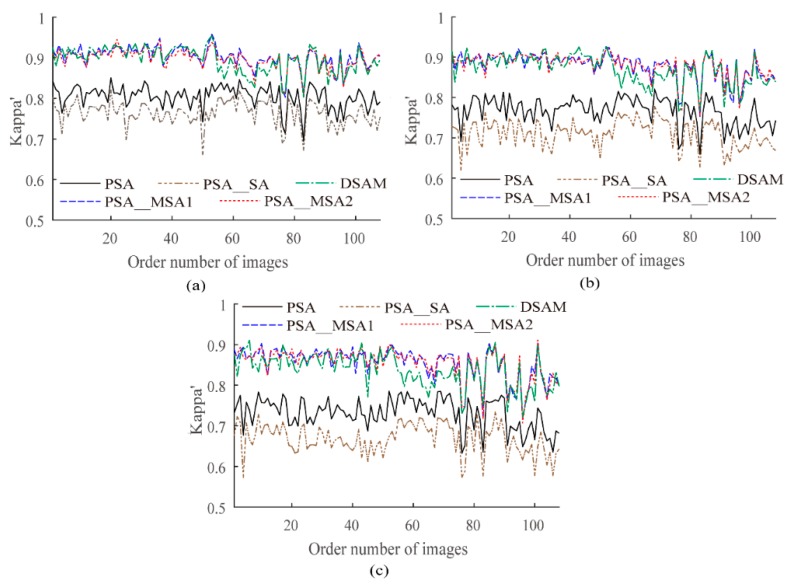
The improved Kappa′ values (%) of different algorithms for the synthetic data set. (**a**) *S* = 8, (**b**) *S* = 10, (**c**) *S* = 12.

To further show that the proposed PSA_MSA algorithm is superior to PSA, PSA_SA, and DSAM, we generated a synthetic data set which contains 108 artificial land images of size 240 × 240 and four kinds of classes, including various shapes. The reconstruction scales were set to 8, 10, and 12. Using PCC′ and Kappa′ as evaluation indexes, the results are plotted as curves in Figure 8 and Figure 9. Equations (6) and (7) are used to compare the accuracies of PSA_MSA1 and PSA_MSA2 with those of PSA, PSA_SA, and DSAM, respectively. In this experiment, *num* = 108.

(6) $$\bar{\Delta {\mathrm{PCC}}^{\prime }}=\frac{\sum_{i=1}^{num}\frac{{\mathrm{PCC}}^{\prime }{}_{i}^{{\mathrm{algorithm}}_{1}}-{\mathrm{PCC}}^{\prime }{}_{i}^{{\mathrm{algorithm}}_{2}}}{{\mathrm{PCC}}^{\prime }{}_{i}^{{\mathrm{algorithm}}_{2}}}\times 100\%}{num}$$

(7) $$\bar{\Delta {\mathrm{Kappa}}^{\prime }}=\frac{\sum_{i=1}^{num}\frac{{\mathrm{Kappa}}^{\prime }{}_{i}^{{\mathrm{algorithm}}_{1}}-{\mathrm{Kappa}}^{\prime }{}_{i}^{{\mathrm{algorithm}}_{2}}}{{\mathrm{Kappa}}^{\prime }{}_{i}^{{\mathrm{algorithm}}_{2}}}\times 100\%}{num}$$

As shown in Table 3 and Table 4, the larger the reconstruction scale is, the better the performance of PSA_MSA compared to those of PSA, PSA_SA, and DSAM. The results further confirmed that PSA_MSA achieves a better accuracy than PSA, PSA_SA, and DSAM in SPM.

### 3.3. Real land Image

In this section, to further demonstrate the advantages of the PSA_MSA algorithm, two real land images are selected as the experimental objects. One is a highly developed area with high spatial autocorrelation and is marked as real land image1, while the other, which is marked as real land image2, is a region with low spatial autocorrelation that has not been highly developed.

**Figure 10:**
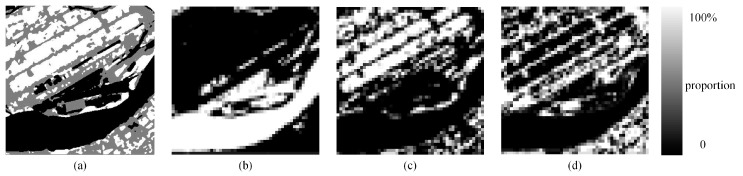
Proportional images of three classes with a scale of 8. (**a**) Reference image, (**b**) water, (**c**) vegetation, (**d**) soil.

Real land image1 is a remote sensing image of Nanjing, Jiangsu Province. The image was taken by the Landsat 8 satellite, which was launched in 2013. The 30-m reference image was taken on October 9, 2017, and contains a total of 396 × 396 pixels. After the image was processed by using K-means classification [33,41], three different classes which shown in Figure 10a were obtained: water, vegetation and soil. Real land image2 is a remote sensing image of Ningbo, Zhejiang Province. The 30-m reference image was taken by the Landsat 8 satellite on August 24, 2017, and contains a total of 398 × 398 pixels. The data can be downloaded from the following website: http://www.gscloud.cn/. The *S* × *S* mean filter was used to degrade these two high-resolution images. The reconstruction scale was set to 8 and 10. The parameters of the SA-based algorithms are mentioned in Section 3.1.

(1) Real land image1

**Figure 11:**
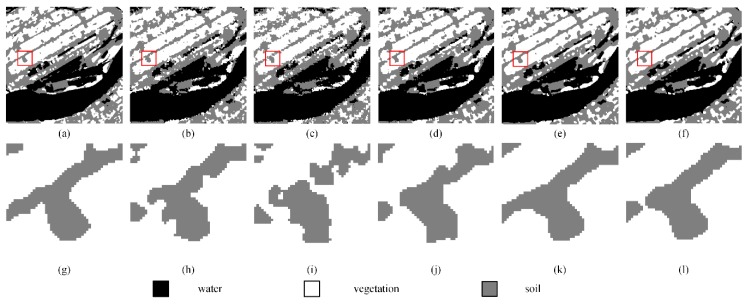
Comparison of the results from different SPM models for a real land image at a reconstruction scale of 8: (**a**) the reference image, (**b**) the PSA results, (**c**) the PSA_SA results, (**d**) the DSAM algorithm results, (**e**) the PSA_MSA1 algorithm results, (**f**) the PSA_MSA2 algorithm results, (**g**) the local details in the reference image, (**h**) the local details in the PSA results, (**i**) the local details in the PSA_SA results, (**j**) the local details in the DSAM algorithm results, (**k**) the local details in the PSA_MSA1 algorithm results, and (**l**) the local details in the PSA_MSA2 algorithm results.

Figure 11a–f display the SPM results of the PSA, the PSA_SA, the DSAM algorithm, the PSA_MSA1 algorithm, and the PSA_MSA2 algorithm at *S* = 8, respectively. Although there were some jagged boundaries for different land covers, the SPM results of the MSA-based algorithms were better than the results of other algorithms. To further demonstrate the superiority of the MSA-based algorithms, a local area was selected and enlarged to show the mapping results. Obviously, the PSA_MSA algorithms reconstructed the target image much more effectively than the other methods.

Table 5 summarizes the SPM results of various models. Both the PCC′ and Kappa′ values from the PSA_MSA1 and PSA_MSA2 algorithms are much better than those values from the other algorithms. Taking *S* = 10 as an example, the improved Kappa′ value from the PSA_MSA1 and PSA_MSA2 algorithms reached 71.42 and 71.44, respectively. The improved Kappa′ value from the PSA_MSA1 algorithm was 11.28%, which is 18.58% higher than that of the PSA and the PSA_SA algorithm. Moreover, it is 4.54% higher than that of DSAM as well. Additionally, the improved Kappa′ value from the PSA_MSA2 algorithm was 11.31%, which is 18.61% higher than that of the PSA and the PSA_SA algorithm. It is 4.57% higher than that of DSAM as well. The PSA_MSA2 algorithm performed slightly better than the PSA_MSA1 algorithm in the case of complex mixing land cover pixels.

The SPM accuracy (improved PCC′) for each land cover type from the five methods is also listed in Table 6. All land covers achieved the highest accuracy levels with the PSA_MSA algorithms.

(2) Real land image2

**Figure 12:**
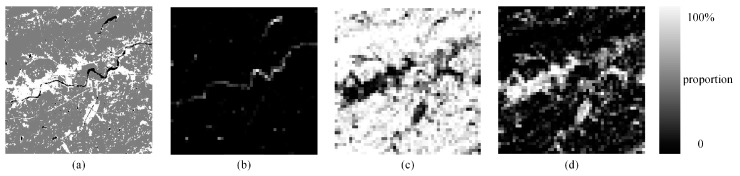
Proportion images of three classes with a scale of 8. (**a**) Reference image, (**b**) water, (**c**) vegetation, (**d**) soil.

**Figure 13:**
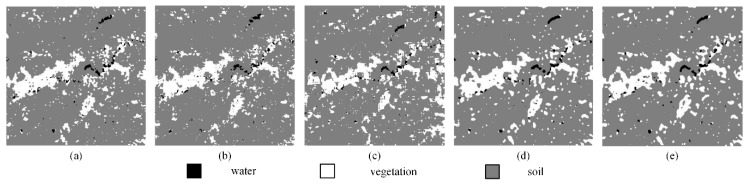
Comparison of the results of different SPM models for an artificial land image with a scale of 8. (**a**) PSA result, (**b**) PSA_SA result, (**c**) DSAM result, (**d**) PSA_MSA1 result, (**e**) PSA_MSA2 result.

We also used the K-means method to obtain three different classes in this image Figure 12a: water, vegetation, and soil. The SPM results of the PSA, the PSA_SA, the DSAM algorithm, the PSA_MSA1 algorithm and the PSA_MSA2 algorithm at *S* = 8 are displayed in Figure 13a–e, respectively. The accuracies of all the algorithms for real land image2 are lower than the result of SPM for real land image1. This shows that these algorithms are more suitable for images with high spatial autocorrelation.

As seen in Table 7, if the land covers in the image are trivial and lack spatial correlation, then the mapping accuracy of all algorithms based on spatial dependence theory will decrease. Although the accuracies of all the algorithms decreased, it can be seen from Table 7 that both the improved PCC′ and Kappa′ values from the PSA_MSA1 and PSA_MSA2 algorithms are still better than the values from the other algorithms. However, it should be pointed out that if the spatial distribution of the image is too scattered, then PSA_MSA will also fail. Thus, for the image with weak spatial correlation, it is necessary to improve the accuracy of proportional image acquisition and introduce variation function to subpixel mapping.

The SPM accuracies (improved PCC′) for each land cover type from the five methods are also listed in Table 8. Vegetation achieved the highest accuracies with the PSA_MSA algorithm.

### 3.4. Influence of Errors on the Process of Proportion Acquisition in the SPM_MSA Algorithm

**Figure 14:**
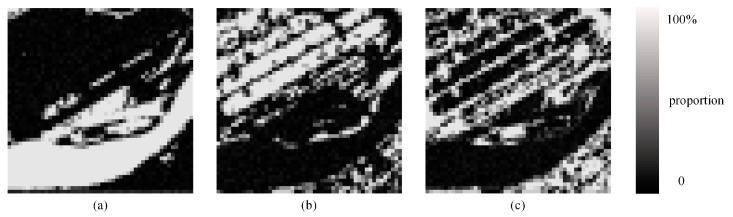
Proportional images of three classes with a 10% error in the proportional image (*S* = 8). (**a**) Water, (**b**) vegetation, (**c**) soil.

**Figure 15:**
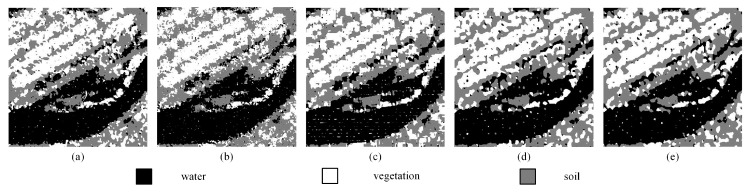
Comparison results of different SPM models for real land image1 with a 10% error in the proportional image (*S* = 10). (**a**) PSA result, (**b**) PSA_SA result, (**c**) DSAM result, (**d**) PSA_MSA1 result, (**e**) PSA_MSA2 result.

It is unavoidable that there will be some errors in the process of obtaining end element proportions caused by spectral unmixing. To further investigate the influence of proportion errors on the PSA_MSA algorithm, we introduce a random error in the process of generating the proportional image, which creates a certain number of errors between the proportional image applied to the PSA_MSA algorithm and the actual proportional image. Real land image1 was selected as the test object. We added 5% and 10% random errors in the proportion image. Figure 14 shows proportion images with an increase of 10% error and containing no pure pixels. The SPM result can be seen in Figure 15. Since the proportion errors are added to each mixed pixel, PCC is equivalent to the improved PCC′ and Kappa is equivalent to the improved Kappa′. Thus, PCC and Kappa are selected as the evaluation indexes for comparison with the case of 0 error. Table 9 and Table 10 summarize the subpixel mapping accuracies of the different algorithms under different errors. It is not difficult to see that the subpixel mapping accuracies of all the algorithms decrease with increasing errors. This shows that errors in the input data will reduce the SPM accuracy. As shown in Figure 15, all the algorithms maximize the spatial correlations of the proportion errors of different land covers existing in the mixed pixels, thereby aggregating them and badly affecting the accuracy of the final subpixel mapping. Table 9 and Table 10 show that the lower the error of the proportion value is, the higher the accuracy of the PSA_MSA algorithm is. This shows the effectiveness of the proposed PSA_MSA algorithm. In other words, if the accuracy of proportion data can be improved, the advantage of PSA_MSA will be more obvious. Compared with PSA and PSA_SA, both DSAM and PSA_MSA can provide SPM results with the best visual and numerical effects.

### 3.5. Stochastic Optimization of Mixed Pixels in the PSA_MSA Algorithm

In step 4 of the proposed PSA_MSA algorithm, we optimized the mixed pixels in a random sequence, which is called stochastic optimization. In traditional optimization, the mixed pixels are sequentially processed by rows or columns, as shown in Figure 4a. Stochastic optimization can change the optimization direction. To analyze the effect stochastic optimization for the PSA_MSA algorithm, the two optimization methods were implemented in the secondary optimization phase of the PSA_MSA algorithm, and the positioning accuracy results were compared. The iteration times for the two optimization methods were the same, and the total optimizations accepted by a single mixed pixel were also the same. Circles and stars were selected as the test objects. Table 11 shows the results after setting the reconstruction scale to 8, and Table 12 shows the results after setting the reconstruction scale to 10. Stochastic optimization can achieve a better SPM accuracy than sequential optimization.

## 4. Discussion

**Figure 16:**
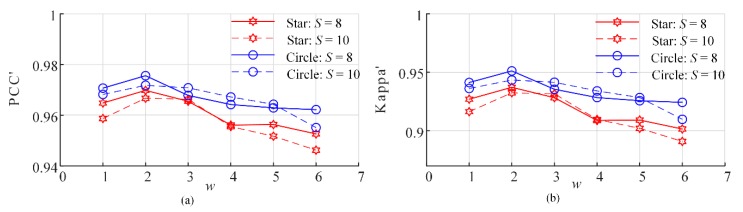
Accuracies of the PSA_MSA1 algorithm under different low attractiveness ranges. (**a**) PCC′ and (**b**) Kappa′.

**Figure 17:**
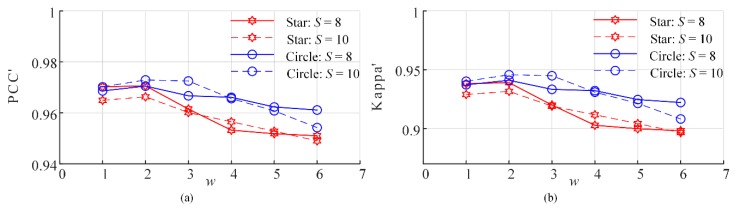
Accuracy of the PSA_MSA2 algorithm under different ranges of low attractiveness. (**a**) PCC′ and (**b**) Kappa′.

The primary different between the PSA_MSA method with the PSA_SA method is that the selection range for the subpixels to be swapped is narrower in the PSA_MSA method. The results shown in Section 3 demonstrate that the SPM positioning accuracy is significantly improved. In Section 3, we randomly selected an M-class subpixel of in the gravitational range of [0, $A_{M_{2}}$] and an N-class subpixel in the gravitational range of [0, $A_{N_{2}}$] for swapping. In fact, other low attractiveness ranges can be selected, such as [0, $A_{M_{3}}$] and [0, $A_{N_{3}}$] or [0, $A_{M_{m}}$] and [0, $A_{N_{n}}$]. To analyze the effect of the low attractiveness range, six different low attractiveness ranges were selected for the PSA_MSA algorithm: from [0, $A_{M_{1}}$] and [0, $A_{N_{1}}$] to [0, $A_{M_{6}}$] and [0, $A_{N_{6}}$], respectively. Therefore, in the experiments, *m* = *n* = *w* (*w*= 1, 2, …,6). Circles and stars were selected as the test objects, and the SPM accuracies are shown in Figure 16 and Figure 17.

The above results demonstrate that the SPM accuracy is better when *w* = 2 or 3, which corresponds to [0, $A_{M_{2}}$] and [0, $A_{N_{2}}$] and [0, $A_{M_{3}}$] and [0, $A_{N_{3}}$], respectively. As the range of low attractiveness widens, the SPM accuracy decreases. However, the optimal range for subpixel swapping might be different in different scenarios. In this research, all the experiments in Section 3 are performed with *w* = 2.

## 5. Conclusions

SPM methods can improve the spatial resolution of spectral images. The traditional PSA model is prone to fall into a local optimum during subpixel swapping. To solve this problem, we proposed a PSA_MSA method that uses SA to randomly swap two subpixels from two different classes. These two subpixels are selected from subpixels with class-specific attractiveness values in certain low ranges. This SA algorithm enables subpixel swapping to escape the local optimum solutions. Only the subpixels with low class-specific attractiveness values may be selected for swapping. Two spatial correlation models were applied to calculate the attractiveness coefficients and compare the mapping results. Experiments were performed with different shapes and land cover images. Compared with other models, such as the PSA, the PSA_SA, and the DSAM algorithm, the proposed PSA_MSA performed better than the others did in the reconstruction of spectral images. The improved PCC′ and Kappa′ values also showed that the proposed PSA_MSA algorithm can generate higher accuracy SPM results than the other algorithms. The PSA_MSA method involved random selection. The experiments also confirmed that a randomized optimization sequence can also improve the mapping accuracy.

The effect of errors in the input proportion images were discussed. The results showed that the larger values of proportion errors lead to low mapping accuracy of SPM algorithms. These proportion errors are mistaken as wrong end-members by the algorithms. SPM algorithms will transform the wrong proportion information of the end-members into spatial information. To reduce the effects of the proportion errors, it is very important to obtain more accurate proportion information in the spectral unmixing procedure. In addition, the PSA_MSA algorithm needs a large number of iterations to obtain global solution. It is meaningful to look for an improved method to shorten the running time of PSA_MSA algorithm. We are considering the following work in the future: First, we aim to study the spectral unmixing algorithms and improve the estimation accuracy to reduce the errors in the proportion images. Second, we aim to further optimize the mapping result, the spectral unmixing procedure, and end-member mapping process while combining all three in an integrated framework. Third, further analysis will be conducted regarding factors that may affect the performance of subpixel mapping algorithms based on spatial dependence theory, such as the spatial resolutions of remote sensed images, the number of end-members in a mixed pixel, and the scattering distribution of the end-members.

## Figures and Tables

**Table 1:** Accuracy results (%) from different models for images with artificial shapes (S = 10).

		PCC	Kappa	Improved PCC′	Improved Kappa′
Circle	PSA	99.56	98.91	90.65	81.25
	PSA_SA algorithm	99.52	98.82	89.80	79.55
	DSAM	99.72	99.31	94.07	88.11
	PSA_MSA1 algorithm	99.91	99.77	98.03	96.05
	PSA_MSA2 algorithm	99.90	99.75	97.88	95.75
Star	PSA	99.33	98.01	89.13	77.91
	PSA_SA algorithm	99.20	97.64	87.10	73.83
	DSAM	99.65	98.96	94.29	88.41
	PSA_MSA1 algorithm	99.88	99.63	97.98	95.91
	PSA_MSA2 algorithm	99.85	99.56	97.56	95.06
Letters	PSA	98.44	87.66	88.25	76.51
	PSA_SA algorithm	98.10	84.91	85.64	71.29
	DSAM	99.20	93.69	93.95	87.89
	PSA_MSA1 algorithm	99.73	97.86	97.94	95.88
	PSA_MSA2 algorithm	99.75	98.00	98.08	96.15

**Table 2:** Accuracy results (%) from different models for an artificial land image.

		PCC	Kappa	Improved PCC′	Improved Kappa′
*S* = 8	PSA	97.64	96.80	91.21	88.15
	PSA_SA algorithm	97.36	96.43	90.17	86.75
	DSAM	98.44	97.89	94.19	92.17
	PSA_MSA1 algorithm	99.10	98.78	96.64	95.48
	PSA_MSA2 algorithm	99.09	98.76	96.59	95.41
*S* = 10	PSA	96.40	95.12	89.32	85.55
	PSA_SA algorithm	95.61	94.06	87.00	82.42
	DSAM	97.77	96.89	93.38	91.05
	PSA_MSA1 algorithm	98.85	98.44	96.58	95.38
	PSA_MSA2 algorithm	98.80	98.38	96.46	95.21
*S* = 12	PSA	95.15	93.43	88.14	84.02
	PSA_SA algorithm	93.46	91.14	84.00	78.45
	DSAM	97.08	96.05	92.86	90.38
	PSA_MSA1 algorithm	98.41	97.85	96.12	94.77
	PSA_MSA2 algorithm	98.48	97.94	96.28	94.99

**Table 3:** Comparison results ($\bar{\Delta {\mathrm{PCC}}^{\prime }}$ %) of different SPM models for the synthetic data set.

Algorithm_1_	Algorithm_2_	*S* = 8	*S* = 10	*S* = 12
PSA_MSA1	PSA	7.54	9.04	10.21
	PSA_SA	11.01	14.12	16.23
	DSAM	0.45	0.67	1.24
PSA_MSA2	PSA	7.21	8.99	10.14
	PSA_SA	10.68	14.07	16.15
	DSAM	0.15	0.63	1.17

**Table 4:** Comparison results ($\bar{\Delta {\mathrm{Kappa}}^{\prime }}$ %) of different SPM models for the synthetic data set.

Algorithm_1_	Algorithm_2_	*S* = 8	*S* = 10	*S* = 12
PSA_MSA1	PSA	12.14	14.82	17.00
	PSA_SA	18.14	23.87	28.14
	DSAM	0.72	1.07	1.95
PSA_MSA2	PSA	11.61	14.73	16.86
	PSA_SA	17.58	23.78	27.98
	DSAM	0.24	0.99	1.83

**Table 5:** Accuracy (%) results of different models for a real land image.

		PCC	Kappa	Improved PCC′	Improved Kappa′
*S* = 8	PSA	89.56	84.33	81.38	70.45
	PSA_SA algorithm	87.61	81.41	77.91	65.00
	DSAM	90.77	86.15	83.55	73.88
	PSA_MSA1 algorithm	91.66	87.49	85.14	76.46
	PSA_MSA2 algorithm	92.03	88.05	85.80	77.49
*S* = 10	PSA	85.59	78.38	77.37	64.18
	PSA_SA algorithm	84.01	76.01	74.89	60.23
	DSAM	87.26	80.89	79.99	68.32
	PSA_MSA1 algorithm	88.51	82.76	81.95	71.42
	PSA_MSA2 algorithm	88.50	82.75	81.95	71.44

**Table 6:** Improved PCC′ values (%) of different land covers for five methods.

*S* = 8	PSA	PSA_SA	DSAM	PSA_MSA1	PSA_MSA2
Water	84.85	82.06	86.61	90.02	90.60
Vegetation	79.80	75.78	82.55	83.75	84.24
Soil	81.25	77.95	83.11	84.29	85.11
*S* = 10	PSA	PSA_SA	DSAM	PSA_MSA1	PSA_MSA2
Water	82.60	78.89	84.48	87.31	87.68
Vegetation	75.91	73.97	78.90	80.17	80.40
Soil	76.50	74.05	78.94	81.29	80.94

**Table 7:** Accuracy (%) results of different models for the real land image.

		PCC	Kappa	Improved PCC′	Improved Kappa′
*S* = 8	PSA	85.90	58.06	79.64	49.94
	PSA_SA algorithm	86.21	53.66	79.77	49.83
	DSAM	87.15	62.12	81.53	54.35
	PSA_MSA1 algorithm	87.25	62.26	81.59	54.94
	PSA_MSA2 algorithm	87.14	61.81	81.43	54.40
*S* = 10	PSA	85.08	55.17	80.96	49.47
	PSA_SA algorithm	85.05	49.10	80.40	47.24
	DSAM	85.58	56.69	81.59	50.90
	PSA_MSA1 algorithm	85.38	56.40	81.33	50.86
	PSA_MSA2 algorithm	85.66	56.95	81.69	51.47

**Table 8:** Improved PCC′ values (%) of different land covers types for the five methods.

*S* = 8	PSA	PSA_SA	DSAM	PSA_MSA1	PSA_MSA2
Water	44.23	37.41	50.38	49.87	48.80
Vegetation	90.02	90.43	90.20	90.91	90.94
Soil	55.91	56.00	58.73	60.28	59.73
*S* = 10	PSA	PSA_SA	DSAM	PSA_MSA1	PSA_MSA2
Water	36.51	30.12	39.72	40.23	40.39
Vegetation	91.23	91.60	90.57	91.22	91.64
Soil	54.49	51.71	57.36	55.73	55.90

**Table 9:** PCC values (%) with different numbers of errors for methods based on PSA.

		PSA	PSA_SA	DSAM	PSA_MSA1	PSA_MSA2
*S* = 8	0	89.56	87.61	90.74	91.66	92.03
	5%	88.23	86.45	89.50	89.81	89.94
	10%	86.22	84.77	88.12	87.82	87.80
*S* = 10	0	85.59	84.01	87.26	88.51	88.50
	5%	84.42	82.88	85.89	86.10	86.63
	10%	82.55	81.35	84.04	84.09	84.57

**Table 10:** Kappa values (%) with different numbers of errors for methods based on PSA.

		PSA	PSA_SA	DSAM	PSA_MSA1	PSA_MSA2
*S* = 8	0	84.33	81.41	86.11	87.49	88.05
	5%	82.35	79.67	84.24	84.71	84.90
	10%	79.32	77.15	82.18	81.72	81.69
*S* = 10	0	78.38	76.01	80.89	82.76	82.75
	5%	76.63	74.31	78.83	79.14	79.94
	10%	73.83	72.01	76.05	76.12	76.85

**Table 11:** Accuracies (%) of two optimization methods for the PSA_MSA1 algorithm.

		Circle		Star	
		Improved PCC′	Improved Kappa′	Improved PCC′	Improved Kappa′
*S* = 8	Sequential	97.63	95.25	96.70	93.15
	Stochastic	97.93	95.86	97.79	95.42
*S* = 10	Sequential	97.89	95.77	97.85	95.64
	Stochastic	98.03	96.05	97.98	95.91

**Table 12:** Accuracies (%) of two optimization methods for the PSA_MSA2 algorithm.

		Circle		Star	
		Improved PCC′	Improved Kappa′	Improved PCC′	Improved Kappa′
*S* = 8	Sequential	97.59	95.17	97.27	94.32
	Stochastic	97.75	95.50	97.31	94.41
*S* = 10	Sequential	97.51	94.99	97.20	94.32
	Stochastic	97.88	95.75	97.56	95.06
